# The Activity Improvement of the TM_3_(hexaiminotriphenylene)_2_ Monolayer for Oxygen Reduction Electrocatalysis: A Density Functional Theory Study

**DOI:** 10.3389/fchem.2018.00351

**Published:** 2018-09-12

**Authors:** Beibei Xiao, Hui Zhu, HouYi Liu, XiaoBao Jiang, Qing Jiang

**Affiliations:** ^1^School of Energy and Power Engineering, Jiangsu University of Science and Technology, Zhenjiang, China; ^2^School of Materials Science and Engineering, Jiangsu University of Science and Technology, Zhenjiang, China; ^3^Key Laboratory of Automobile Materials, Ministry of Education, School of Materials Science and Engineering, Jilin University, Changchun, China

**Keywords:** oxygen reduction reaction, activity and selectivity, 2D materials, transition metal elements, DFT calculation

## Abstract

Polymer electrolyte membrane fuel cells (PEMFCs) are one of the most prominent clean energy technologies designed to achieve hydrogen utilization and solve problems such as low efficiency and high pollution associated with fossil fuel combustion. In order to bring about PEMFC commercialization, especially for automobile applications, developing high-activity and -selectivity catalysts for the oxygen reduction reaction (ORR) is of critical importance. Based on the density functional theory, the catalytic activity of the conductive, two-dimensional metal–organic frameworks TM_3_(HITP)_2_ monolayer (where HITP = hexaiminotriphenylene; TM = Ni, Co, Fe, Pd, Rh, Ru, Pt, Ir, and Os) for ORR has been investigated systematically. Furthermore, the classical volcano curves of the ORR activity, as a function of the OH binding, are found where the Ni, Pd, and Pt located at the weak binding side suffer from the sluggish ^*^OOH formation and prefer the inefficient 2*e*^−^ mechanism, while for other elements belonging to the strong binding side, the reactions are hindered by the poison due to ORR intermediates. Based on the free energy profiles, the corresponding overpotentials μ_ORR_ exhibit the inverted volcano curve as a function of the atomic number of the 3*d*/4*d*/5*d* TM active center in the same period. Based on the μ_ORR_ data, ORR activity decreases in the order of Ir > Co ≈ Rh > Ni ≈ Pd > Pt ≈ Fe > Ru > Os. Herein, the Co, Rh, and Ir central atoms exhibit enhanced catalytic activity in combination with the desirable selectivity of the O_2_ reduction to H_2_O. This systematic work may open new avenues for the development of high-performance non-PGM catalysts for practical applications of ORR.

## Introduction

Hydrogen is a potential candidate for future energy provision, as stated in the concept of the hydrogen economy, so as to solve the issues of the rising global energy demands, depletion of fossil fuel reserves, and associated environmental pollution issues. Due to its high efficiency, ease of operation, and low emission, the polymer electrolyte membrane fuel cell (PEMFCs) is the most prominent technology to derive benefit from the proposed hydrogen cycle, which leads to the production of electricity from the electrochemical oxidation of hydrogen, with water as its endproduct (Colić and Bandarenka, 2016; Xia Z. et al., 2016; Chen et al., 2017, 2018). However, a critical obstacle to its commercialization is the dominant voltage loss associated with the sluggish oxygen reduction reaction (ORR), even when catalyzed by the noble Pt nanoparticle (Nørskov et al., 2004). In this regard, a significant amount of Pt is required to achieve the desirable power density, making the overall cost prohibitively high (Debe, 2012). In order to overcome the economic bottleneck, design, and application of earth-abundant alternatives for ORR electrocatalysis are at the heart of PEMFC research (Xia W. et al., 2016).

Inspired by the pioneering work on cobalt phthalocyanines acting as cathode catalysts (Jasinski, 1964), a tremendous amount of research is being performed on the TM–N_x_ carbon materials for ORR (TM denotes transition metals), especially TM–N_4_ active motif. The intrinsic active characteristic of TM in N_x_ carbon materials is experimentally supported, where the presence of the TM atom would boost the ORR performance compared with the metal-free counterpart (Peng et al., 2013; Yin et al., 2016; Yang et al., 2017); the corresponding activity would be suppressed by adding the SCN^−^ ions and CO molecule (Zhang et al., 2016; Yang et al., 2017). Linear correlations between the content of TM–N_x_ and ORR activity have been observed (Yang et al., 2017). Furthermore, ORR activity shows its dependence on the TM center atom, and this is supported by the density functional theory (DFT), a theoretical work of Rossmeisl et al., where the ORR activity of the TM–N_4_ embedded graphene has been systematically investigated, and where the Fe, Ir, Mn, Ru, and Rh doping is identified as boosting the ORR (Calle-Vallejo et al., 2011). Additionally, the ORR mechanism is sensitive to the TM–N_4_ active motif. Liu et al. have synthesized the Fe–N_x_ and Co–N_x_ doped carbon nanofiber and realized that the Fe–N_4_ promotes 4*e*^−^ ORR in comparison with the 2*e*^−^ pathway of the Co–N_4_ one (Liu et al., 2016). The same conclusion has been achieved by Kattel et al., where the O–O bond scission is presented and the efficient 4*e*^−^ reduction of O_2_ to 2H_2_O is preferred on the Fe–N_4_ sites, but the reduction of O_2_ to H_2_O_2_ is found enhanced on the Co–N_4_ motif (Kattel et al., 2013, 2014).

Despite these encouraging research works, such TM–N_4_ carbon materials generally suffer from low activity caused by the relatively few catalytic sites as well as the experimental challenge of the well-controlled active motif (Liang et al., 2013; Peng et al., 2013; Palaniselvam et al., 2016), reducing the competition with the state-of-the-art Pt/C catalysts. Paying attention to the high TM–N_4_ density in combination with the electronic conductivity, Miner et al. have developed the attractive Ni_3_(HITP)_2_ as ORR electrocatalyst (Miner et al., 2016). However, the performance is far from satisfying expectations, with its incomplete oxidation of O_2_ and the predominant production of H_2_O_2_ under the working potential (Miner et al., 2016). In this regard, utilizing the aforementioned information, the development of the efficient TM_3_(HITP)_2_ catalysts can be achieved via the variation of metal nodes (Choi et al., 2015; Zhang et al., 2015). It has naturally raised the interest to search for the optimal combinations of the TM_3_(HITP)_2_, possessing superior ORR activity as well as selectivity.

**Figure 1:**
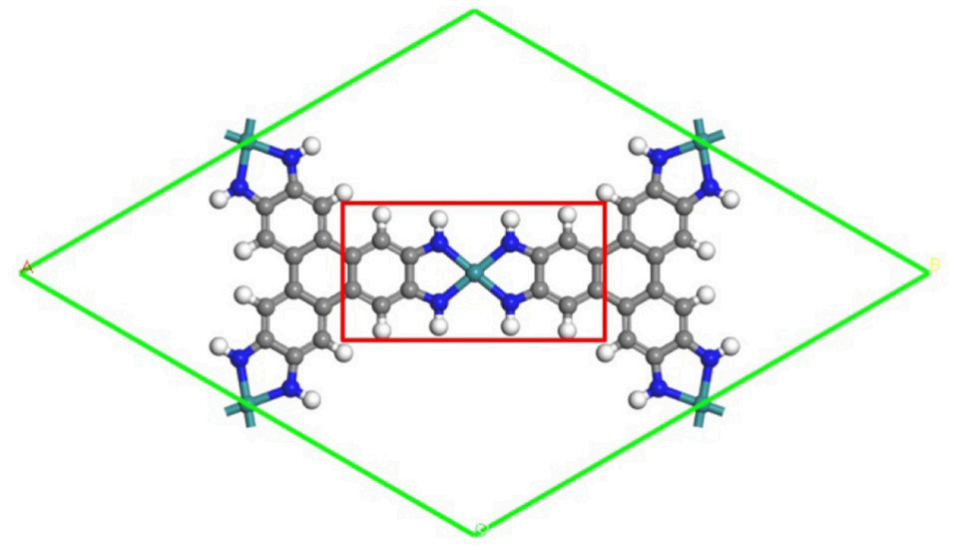
The schematic structure of the TM_3_(HITP)_2_ monolayer. Green, blue, gray, and white denote the TM, N, C, and H atoms.

In the manuscript, DFT calculations are used within an electrochemical framework to analyze the ORR electrocatalysis over the TM_3_(HITP)_2_monolayer. In particular, the stability of the ORR intermediates is calculated, which allows to evaluate the thermodynamic ORR free energy and its overpotentials. The data provide the fundamental understanding of the mechanism of Ni_3_(HITP)_2_ and further identify optimal candidates as catalysts. According the *d*–partial density of states, an atomistic insight of the activity origin has been provided by a thorough comparison among the considered systems. Herein, for simplification, our attention is mainly focused on the monolayer structure shown in Figure 1 as a representative model due to the weak interaction between the interlayers of the 2D-layered materials (Sheberla et al., 2014; Chen et al., 2015; Miner et al., 2016).

## Methods

All calculations have been performed within the DFT framework, as implemented in the DMol^3^ code (Delley, 1990, 2000). The generalized gradient approximation with the Perdew–Burke–Ernzerhof (PBE) functional is employed to describe exchange and correlation effects (Perdew et al., 1996). The DFT semicore pseudopotentials (DSPP) core treat method is implemented for relativistic effects, which replaces core electrons by a single effective potential and introduces some degree of relativistic correction into the core (Delley, 2002). The double numerical atomic orbital augmented by a polarization function (DNP) is chosen as the basis set (Delley, 1990). A smearing of 0.005 Ha (1 Ha = 27.21 eV) to the orbital occupation is applied to achieve accurate electronic convergence. In order to ensure high-quality results, the real-space global orbital cutoff radius is set as high as 5.2 Å. In order to accurately describe the long-range electrostatic interactions of the ORR intermediates with catalysts, the PBE-D method with the TS van der Waals (vdW) correction is employed. In the geometry optimization of structures, the convergence tolerances of energy, maximum force, and displacement are 1.0 × 10^−5^ Ha, 0.002 Ha/Å, and 0.005 Å, respectively. The spin-unrestricted method is used for all calculations. A conductor-like screening model (COSMO) was used to simulate a H_2_O solvent environment for all calculations (Todorova and Delley, 2008), which is a continuum model, where the solute molecule forms a cavity within the dielectric continuum. The DMol^3^/COSMO method has been generalized to periodic boundary cases. The dielectric constant is set as 78.54 for H_2_O. Some previous results have shown that this implicit solvation model is an effective method to describe solvation (Sha et al., 2011; Zhang et al., 2015). The 15 Å-thick vacuum is added to avoid the artificial interactions between the TM_3_(HITP)_2_ monolayer and its images. The proposed structure of the TM_3_(HITP)_2_ monolayer is shown in Figure 1, where the atoms in the red square are fixed in all of the structure-optimization calculations.

The adsorption energy (*E*_ads_) of the reactant O_2_ is calculated by the following,

(1) $$\begin{array}{r} {\text{E}}_{\text{ads}}\left({\text{O}}_{2}\right)={\text{E}}_{\text{sys}}-{\text{E}}_{\text{substrate}}-{\text{E}}_{\text{O}2} \end{array}$$

The adsorption energy (*E*_ads_) of the ORR intermediates are calculated relative to H_2_O and H_2_ (Calle-Vallejo et al., 2011), through,

(2) $$\begin{array}{rcl} {\text{E}}_{\text{ads}}\left(\text{OOH}\right) & = & {\text{E}}_{\text{sys}}-{\text{E}}_{\text{substrate}}-\left(2{\text{E}}_{\text{H}2\text{O}}-3/2{\text{E}}_{\text{H}2}\right) \end{array}$$

(3) $$\begin{array}{rcl} {\text{E}}_{\text{ads}}\left(\text{O}\right) & = & {\text{E}}_{\text{sys}}-{\text{E}}_{\text{substrate}}-\left({\text{E}}_{\text{H}2\text{O}}-{\text{E}}_{\text{H}2}\right) \end{array}$$

(4) $$\begin{array}{rcl} {\text{E}}_{\text{ads}}\left(\text{OH}\right) & = & {\text{E}}_{\text{sys}}-{\text{E}}_{\text{substrate}}-\left({\text{E}}_{\text{H}2\text{O}}-1/2{\text{E}}_{\text{H}2}\right) \end{array}$$

where *E*_sys_, *E*_substrate_, *E*_H2O_, and *E*_H2_ are the total energy of the adsorption systems, the TM_3_(HITP)_2_ monolayer, H_2_O molecule, and H_2_ molecule, respectively. The *E*_ads_ < 0 corresponds to an exothermic adsorption process.

The Gibbs free energy changes (Δ*G*) of the ORR elemental steps have been calculated according to the computational hydrogen electrode (CHE) model developed by Nørskov et al., where the chemical potential of the proton/electron (H^+^ + *e*^−^) in solution is equal to the half of the chemical potential of a gas-phase H_2_ at the reference relative hydrogen electrode (RHE) potential (Nørskov et al., 2004). Herein, *G*_(H++e−)_ = 1/2*G*_(H2)_. The variance of the proton–electron pair free energy with potential is simply determined using the linear free energy dependence of the electron energy on potential, shifting the electron energy –*eU*, where *e* is the elementary positive charge and *U* is the electrode potential of interest on the RHE scale (Nørskov et al., 2004; Nie et al., 2014). The total chemical potential of a proton–electron pair at the potential *U* is written as follows:

(5) $$\begin{array}{r} \text{G}{\left(\text{U}\right)}_{\left(\text{H}++\text{e}-\right)}=1/2{\text{G}}_{\left(\text{H}2\right)}-\text{eU} \end{array}$$

Therefore, for a general electrochemical reaction, the free energy change Δ*G* for every elemental step can be determined as following:

(6) $$\begin{array}{r} \Delta \text{G}=\Delta \text{E}+\Delta \text{ZPE}-\text{T}\Delta \text{S}+\Delta {\text{G}}_{\text{pH}}+\Delta {\text{G}}_{\text{U}} \end{array}$$

where Δ*E* is the electronic energy difference based on DFT calculations, Δ*ZPE* is the change in zero point energy, *T* is the temperature (equal to 298.15 K here), Δ*S* is the change in the entropy, and Δ*G*_pH_ and Δ*G*_U_ are the free energy contributions due to variation in pH value and electrode potential *U*, respectively. Δ*G*_U_ = –*eU*, in which *U* is the potential related to the standard hydrogen electrode. Δ*G*_pH_ = –*kT*ln10 x pH, which is the corrected free energy of H^+^ ions depending on the concentration. According to the previous works, pH is set as 0 in acid medium and 14 in alkaline medium. The zero-point energies and entropies of the ORR intermediates are calculated from the vibrational frequencies according to standard methods. Following the suggestion of Wilcox et al. (Lim and Wilcox, 2012), in order to reduce the calculation, the TM_3_(HITP)_2_ monolayer is fully constrained. The Δ*G* < 0 corresponds to an exothermic adsorption process. The free energy *G* of O_2_ is derived as *G*(O_2_) = 4.92+2*G*(H_2_O)−2*G*(H_2_) by utilizing OER equilibrium at the standard conditions; the *G* of H_2_O_2_ is derived similarly considering that the H_2_O_2_/O_2_ standard equilibrium potential is 0.682 V vs. SHE (Sun et al., 2014). The CHE model has been successfully applied for developing the novel electrocatalysts with prominent ORR performances, where the DFT calculations are in line with the experimental results (Nørskov et al., 2004; Greeley et al., 2009; Favaro et al., 2015; Lang et al., 2015; Jia et al., 2016; Tang et al., 2016; Liu et al., 2017; Li et al., 2018; Xu et al., 2018). Furthermore, the PBE/DNP method in Dmol^3^ code has been widely employed for evaluating the potential of the TM-based carbon electrocatalysts (Wang et al., 2015, 2016b; Hou et al., 2016; Xiao et al., 2017). Therefore, the reliability of the mentioned approach is confirmed.

## Results and discussion

**Figure 2:**
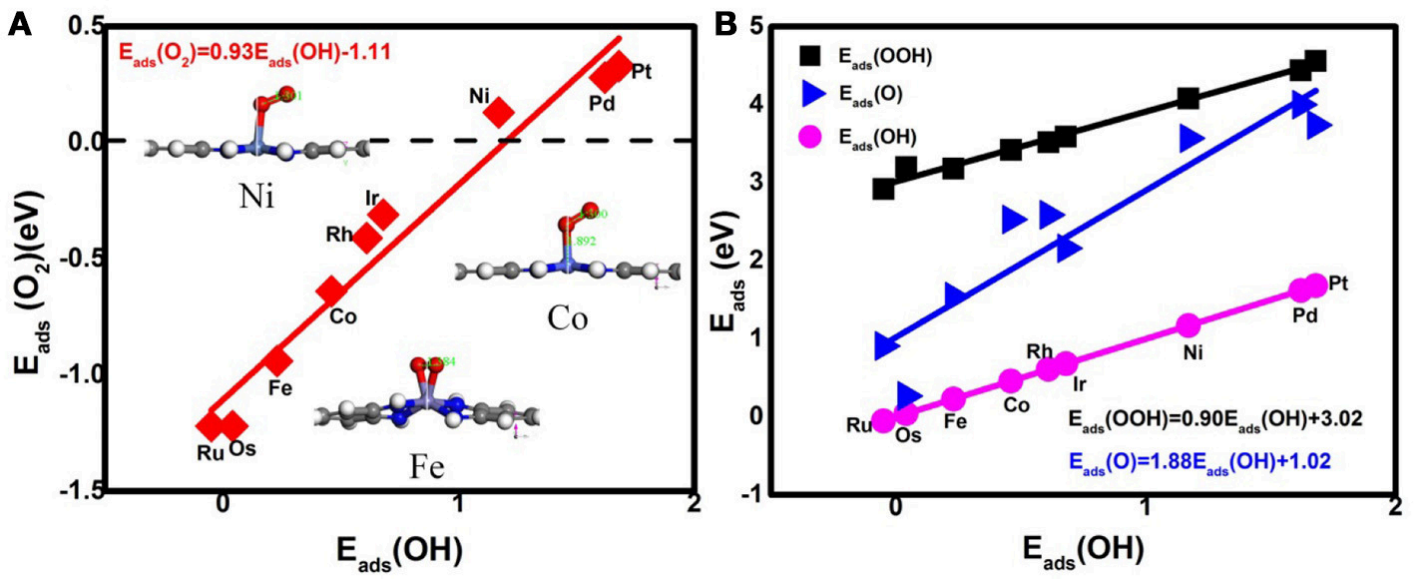
The adsorption energies of the O_2_ molecules **(A)** and the ORR intermediates **(B)**. Inset: the representative O_2_ adsorption structure on Ni, Co, and Fe active centers.

Prior to the investigation of the activity, the essential step is to determine the adsorption behavior of the ORR intermediates. For Ni_3_(HITP)_2_, the energetics of the O_2_ adsorption is endothermic with the value of 0.13 eV, caused by the structure deformation as shown in the inset of Figure 2A, indicating no such adsorption has occurred on the Ni center atom. Besides the reactant, the corresponding *E*_ads_ of ORR intermediates are 4.08, 3.57, and 1.17 eV for OOH, O, and OH, respectively. In comparison with the Pt(111) (3.55 eV for OOH, 1.38 eV for O, and 0.64 eV for OH) (Xiao et al., 2016), the obviously weak adsorption ability of the center Ni atom has been observed.

**Figure 3:**
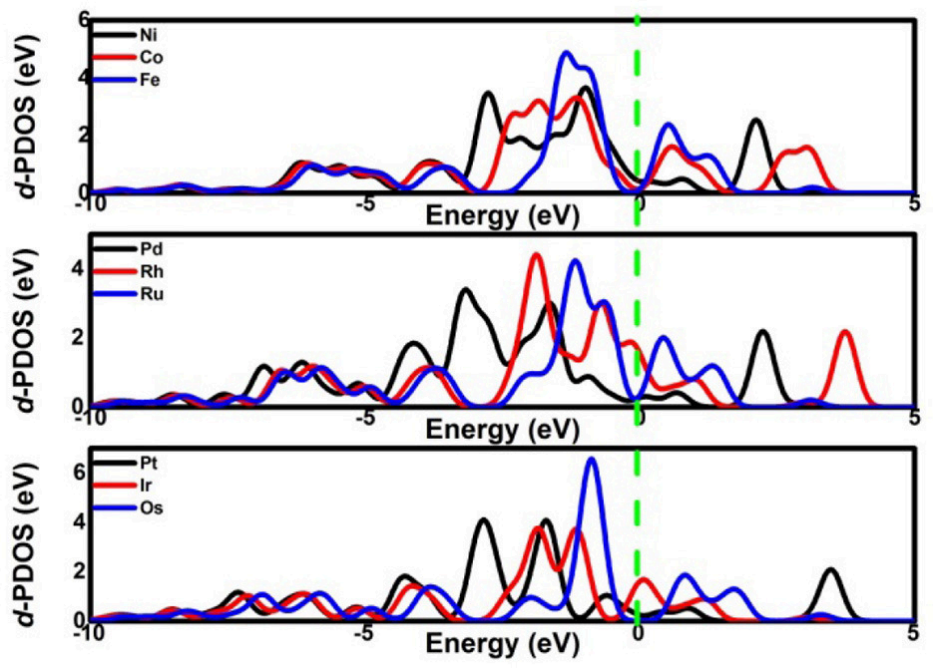
The *d*-PDOS of the central TM atom.

Considering the low affinity of the ORR intermediates to the Ni center, it is expected that the ORR activity would be boosted by selecting suitable TMs. Indeed, the change of the TM center definitely exhibits different adsorption behaviors, as shown in Figure 2, where the corresponding *E*_ads_ data are given. From the figures, the *E*_ads_ of the reactant O_2_ and the corresponding ORR intermediates decrease monotonically in the order of Ru ≈ Os > Fe > Co > Rh ≈ Ir > Ni > Pd ≈ Pt. It is obvious that the adsorption ability of the elements is weakened from group 8 to group 10. Carefully reviewing the O_2_ adsorption as shown in the inset of Figure 2A, similar to Ni, no such adsorption behaviors have occurred at the Pd and Pt active centers due to the endothermic adsorption energies. The unfavorable adsorption behavior of group 10 implies the underlying mechanism of the OOH formation, which originates from the long-range electron transfer from the catalyst to O_2_ molecules at the outer Helmholtz plane (Choi et al., 2014). On the contrary, group 9 binds to the reactant suitably with the corresponding end-on structure and group 8 possesses a too strong O_2_ adsorption with the side-on adsorption configuration. Herein, the mentioned adsorption capability is in line with the general prediction of the *d*-band model (Hammer and Nørskov, 2000). The corresponding evidences are provided by the *d*–partial density of states (*d*–PDOS) of the central TM atom plotted in Figure 3. As shown, the *d* band moves away from the Fermi energy as the TM atom changes from group 8 to group 10. The TM atom with the higher *d* states possesses stronger adsorption ability. Furthermore, due to the relationship between the adsorption behavior and the *d* band, the linear scaling relations between *E*_ads_(O_2_)/*E*_ads_(OOH)/*E*_ads_(O) and *E*_ads_(OH) are observed, in accordance with the previous reports of other functionalized carbon materials (Calle-Vallejo et al., 2011; Baran et al., 2014). That is,

(7) $$\begin{array}{rcl} {\text{E}}_{\text{ads}}\left({\text{O}}_{2}\right) & = & 0.93{\text{E}}_{\text{ads}}\left(\text{OH}\right)-1.11, \end{array}$$

(8) $$\begin{array}{rcl} {\text{E}}_{\text{ads}}\left(\text{OOH}\right) & = & 0.90{\text{E}}_{\text{ads}}\left(\text{OH}\right)+3.02, \end{array}$$

(9) $$\begin{array}{rcl} {\text{E}}_{\text{ads}}\left(\text{O}\right) & = & 1.88{\text{E}}_{\text{ads}}\left(\text{OH}\right)+1.02. \end{array}$$

To further evaluate the ORR activity of TM_3_(HITP)_2_, according to the experimental condition (Miner et al., 2016), the OOH associative mechanisms in the alkaline solution are taken into consideration, with the elemental steps R_*i*_ listed as follows (Wang et al., 2016a), where asterisks denote active TM sites. Due to the small barrier of proton transfer, which can be ignored at high applied voltages, our attention is only focused on the reaction energies (Nørskov et al., 2004; Calle-Vallejo et al., 2011; Zhang et al., 2015).

$$\begin{array}{l} \;{\text{O}}_{\text{2}}(\text{g})+*+{\text{H}}_{\text{2}}\text{O}(\text{l})\text{+}e^{\text{-}}\to {\text{OOH}}^{*}{\text{+OH}}^{\text{-}},\;({\text{R}}_{\text{1}})\\ {\text{OOH}}^{*}+e^{-}\to {\text{O}}^{*}{\text{+OH}}^{\text{-}},\;({\text{R}}_{\text{2a}})\\ {\text{OOH}}^{*}+{\text{H}}_{\text{2}}\text{O}(\text{l})\text{+}e^{\text{-}}\to {\text{H}}_{\text{2}}{\text{O}}_{\text{2}}^{*}{\text{+OH}}^{\text{-}},\;({\text{R}}_{\text{2b}})\\ \;{\text{O}}^{*}+{\text{H}}_{\text{2}}\text{O}(\text{l})\text{+}e^{\text{-}}\to {\text{OH}}^{*}{\text{+OH}}^{\text{-}},\;({\text{R}}_{\text{3}})\\ \;{\text{OH}}^{*}+e^{\text{-}}\to {\text{OH}}^{\text{-}}.\;({\text{R}}_{\text{4}}) \end{array}$$

**Figure 4:**
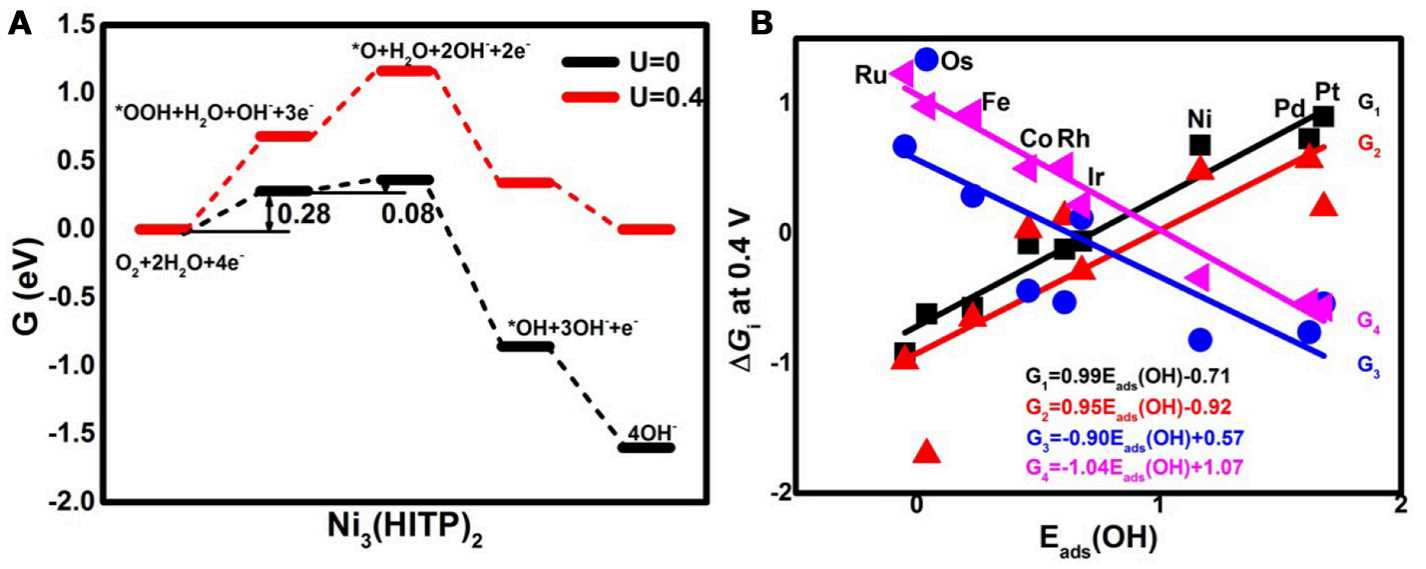
The free energy *G* diagrams of Ni_3_(HITP)_2_ in the alkaline medium **(A)** and the free energy change Δ*G*_i_ of the elemental step R_i_ at the ideal potential of 0.4 eV **(B)**.

Analyzing the free energy plots of the complete 4*e*^−^ ORR pathway in Figure 4A, the endothermic processes of ^*^OOH formation (R_1_) and ^*^O formation (R_2a_) are observed even at *U* = 0 V. The corresponding values of the free energy change Δ*G*_*i*_ are 0.28 and 0.08 eV, respectively. The positive values indicate that the mentioned steps are thermodynamically unfavorable (Nørskov et al., 2004). When the ideal potential of 0.4 eV is applied, the mentioned reaction steps are even unfavorable with the increased Δ*G*_*i*_ of 0.68 and 0.48 eV, respectively. Based on the information, R_1_ is determined as the rate-determining step (RDS), pointing to the fact that the Ni_3_(HITP)_2_ monolayer suffers from insufficient O_2_ activation (Greeley et al., 2009), in accordance with the endothermic capture of the O_2_ molecule as shown in Figure 2A. To clarify the selectivity of the 2/4*e*^−^ mechanism, the comparison between R_2a_ and R_2b_ is considered. In the alternative 2*e*^−^ pathway, the Δ*G*_2b_ of the H_2_O_2_ formation (R_2b_) is increased from 0.01 to 0.41 eV as the *U* ranged from 0 to 0.4 V, being 0.07 eV lower than Δ*G*_2a_, indicating the slightly energy favorable condition of R_2b_. Herein, the predominant production of H_2_O_2_ would be expected on the Ni_3_(HITP)_2_ monolayer, which is in consistence with the experiment results of the 3D Ni_3_(HITP)_2_. In summary, the Ni_3_(HITP)_2_ monolayer slightly prefers the 2*e*^−^ mechanism, with the RDS located at the ^*^OOH formation.

Owing to the different adsorption abilities of TM_3_(HITP)_2_, the suitable TM would boost the ORR activity as well as the selectivity of the 4*e*^−^ mechanism. In order to characterize the relationship between the ORR activity and the TM center atom, the reactive free energy change Δ*G*_*i*_ of the elementary steps R_*i*_ at *U* = 0.4 V as a function of *E*_ads_(OH) is plotted in Figure 4B. As the weakening of the *E*_ads_(OH) occurs with the values ranging from −0.05 to 1.68 eV, Δ*G*_1_ and Δ*G*_2a_ increase from the negative to the positive values, while the opposite tendency is found for Δ*G*_3_ and Δ*G*_4_, which decrease from the positive to the negative values. That is, the steps R_1_ and R_2a_ change from the exothermic to endothermic process, while the steps R_3_ and R_4_ become more thermodynamically favorable. Obviously, the RDS steps are identified as R_4_ for group 8 and group 9 with the exception of Os, where the RDS step is R_3_, while the RDS step is located at R_1_ for group 10.

**Figure 5:**
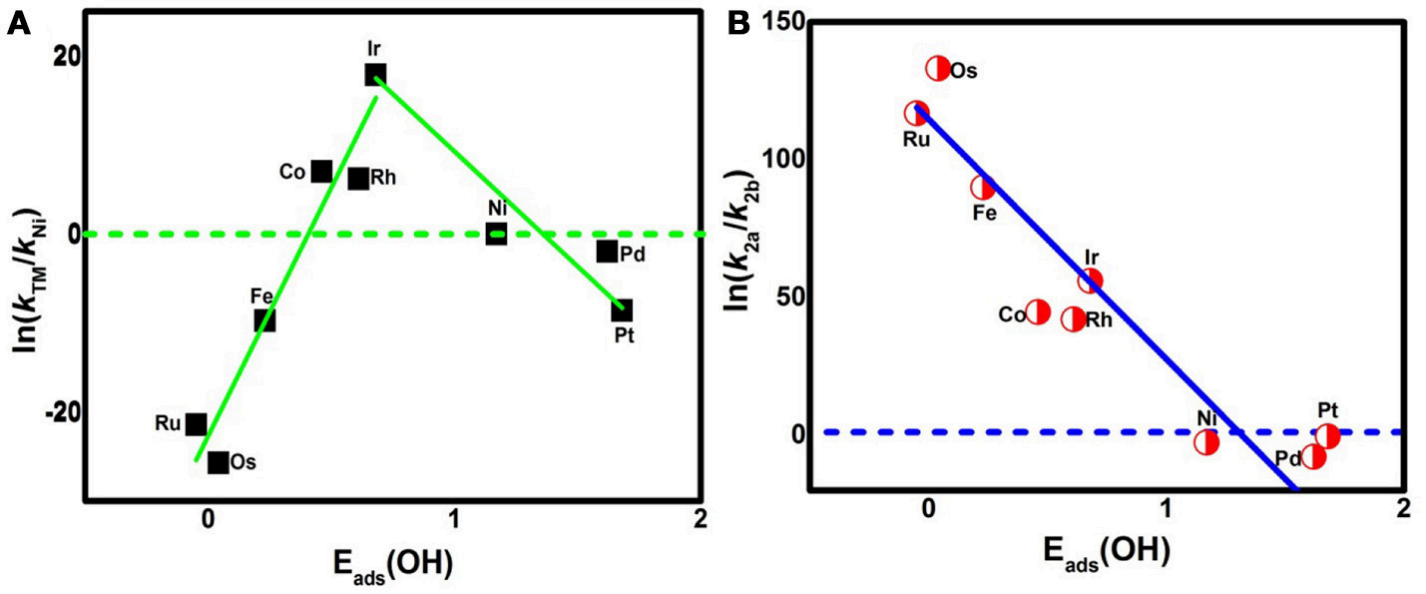
The activity enhancement factor of TM_3_(HITP)_2_ referred to the Ni_3_(HITP)_2_ **(A)** and the selectivity of the 4*e*^−^ reduction referred to the 2*e*^−^ pathway **(B)**.

**Figure 6:**
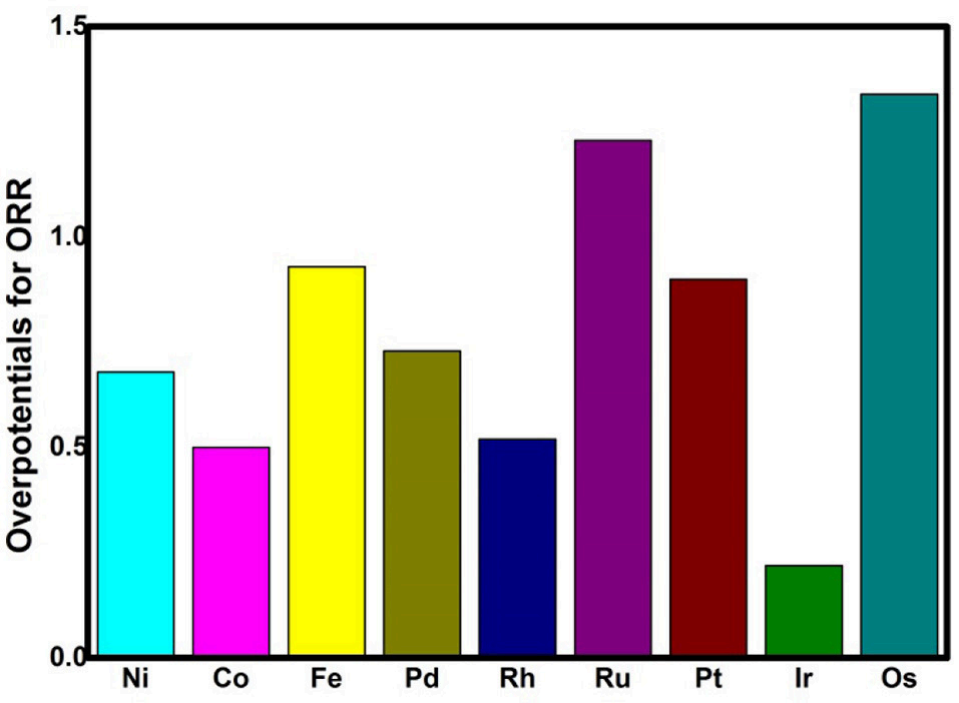
The overpotentials μ_ORR_ of the TM_3_(HITP)_2_ monolayer for the 4*e*^−^ mechanism.

On the basis of the assumption that the activation barrier for the RDS is equal to Δ*G*_max_, the activity variation of TM_3_(HITP)_2_ referred to as Ni_3_(HITP)_2_ is estimated by the rate constant *k* via the simple Arrhenius equation in the following,

(10) $$\begin{array}{rcl} \text{k} & = & \text{Aexp}\left(-\Delta {\text{G}}_{\text{max}}/{\text{k}}_{\text{B}}\text{T}\right) \end{array}$$

(11) $$\text{In}(k_{\text{TM}}/k_{\text{Ni}})\;=[\Delta G_{\text{max}}(\text{Ni})-\Delta G_{\text{max}}(\text{TM})]/k_{\text{B}}\text{T})$$

where A is the prefactor, *k*_B_ is the Boltzmann constant, and T is the temperature (298.15 K). The classic volcano curve is observed in Figure 5A, where the In(*k*_TM_/*k*_Ni_) as a function of the *E*_ads_(OH) is plotted. Based on our results, the adsorption ability is weakened as the atomic number increases from group 8 to group 10. The Ni/Pd/Pt active centers suffer from the too weak binding strength, leading to the energetically unfavorable process of the ^*^OOH formation. Meanwhile, for the Fe/Ru/Os active centers, the too strong interaction with the ORR intermediates accounts for the poisoning of the O-containing intermediates. The suitable binding strength of Co/Rh/Ir active centers indicates the balance between the O_2_ activation and the catalyst recovery. As discussed by the previous reports (Calle-Vallejo et al., 2011; Viswanathan et al., 2012a; Baran et al., 2014; Zhang et al., 2015), the ORR activity depends on the adsorption of the intermediates. Our results are in accordance with the previous reports in that the bond strength should be compromised in the case of the effective ORR catalysts on the basis of Sabatier principle (Greeley et al., 2009; Gao et al., 2017, 2018); this is similar with the previous studies on metal (Stephens et al., 2012), functional graphene (Calle-Vallejo et al., 2011), metal porphyrine (Baran et al., 2014) as well as the 2D MOF (Gao et al., 2017). Herein, in comparison with the Ni_3_(HITP)_2_ monolayer (0.68 eV), group 9 possesses superior catalytic performance with the smaller Δ*G*_max_ of 0.50, 0.52, and 0.22 eV for the Co, Rh, and Ir center atoms, respectively. By contrast, inferior activities are observed for group 8 and group 10. The corresponding Δ*G*_max_ are 0.93, 1.23, and 1.34 eV for Fe, Ru, and Os atoms, and 0.72 and 0.90 eV for Pd and Pt atoms, respectively. Furthermore, for clarification, the data of the relative overpotentials μ_ORR_ are summarized and collected for the activity comparison, depicted in Figure 6. As the atomic number of TM increased from Ni to Fe, the μ_ORR_ reduces and then increases, presenting further evidence to the presence of the classical volcano-shaped activity (Nørskov et al., 2004; Calle-Vallejo et al., 2011; Baran et al., 2014; Zheng et al., 2017). Similar situations are found for the 4*d*/5*d* TM_3_(HITP)_2_ monolayer. Based on the μ_ORR_ data, ORR activity decreases in the order of Ir > Co ≈ Rh > Ni ≈ Pd > Pt ≈ Fe > Ru > Os. Furthermore, compared with the data of the TM supported on graphene and macrocyclic molecules (the minimum μ_ORR_ = 0.4 eV) (Xu et al., 2018), the prominent improvement of the ORR activity on the Ir_3_(HITP)_2_ monolayer is confirmed.

Besides the ORR activity, the ORR selectivity is characterized by the ratio of the corresponding reaction rate *k*_2a_ (*k*_2b_) for step R_2a_ (R_2b_), via the following equation,

(12) $$\text{In}(k_{\text{2a}}/k_{\text{2b}})=[\Delta G_{\text{2b}}-\Delta G_{\text{2a}}]/k_{\text{B}}\text{T})$$

The data are plotted in Figure 5B. As shown, In(*k*_2a_/*k*_2b_) shows a linear relationship with *E*_ads_(OH). For the weak side, the values of Δ*G*_2a_ and Δ*G*_2b_ are 0.48 and 0.41 eV for Ni, 0.57 and 0.37 eV for Pd, and 0.20 and 0.19 eV for Pt, respectively. The corresponding In(*k*_2a_/*k*_2b_) are −2.72, −7.78, and −0.39, indicating the energy favorability of R_2b_ compared with that of R_2a_ for group 10. Furthermore, considering the In(*k*_2a_/*k*_2b_) data, the 2*e*^−^ reduction of O_2_ to H_2_O_2_ is prevalent at the Ni and Pd centers, while the mixing of 2/4*e*^−^ ORR is reasonable for the Pt center. By contrast, R_2a_ is preferred for other elements indicated by the positive In(*k*_2a_/*k*_2b_). That is, the 4*e*^−^ mechanism is dominant for group 8 and group 9. Obviously, as the *E*_ads_(OH) is strengthened, the catalytic selectivity is changed from the 2*e*^−^ to 4*e*^−^ pathway. The two sides of the catalytic activity profile shown in Figure 5A essentially distinguish the 2*e*^−^ catalysts (weak binding side) from the 4*e*^−^ catalysts (strong binding side) (Viswanathan et al., 2012b; Zagal and Koper, 2016). That is, the former suffering from the insufficient O_2_ activation favors the H_2_O_2_ formation; the latter poisoned by the O–containing intermediates generally prefers the H_2_O formation. The formation of H_2_O_2_ not only decreases the ORR efficiency, but also degrades the proton exchange membrane (Tsuneda et al., 2017). In short, the dramatic enhancements in oxygen-reduction rates and product selectivity are achieved by selecting TM centers in group 9, in line with the previous works (Gao et al., 2017; Wannakao et al., 2017).

To understand the influence of the long-term electrostatic potential on the ORR performance, the vdW correction is further applied for analyzing the optimum Ir/Co/Rh systems, where the corresponding adsorption energy and the free energy changes are listed in Table 1. Intuitively, the adsorption abilities of the mentioned systems are enhanced due to the presence of the long-term interaction, in consistence with our calculation data. For the Ir_3_(HITP)_2_ monolayer, the *E*_ads_ are slightly increased to −0.39, 3.49, 2.11, and 0.61 eV for O_2_, OOH, O, and OH, respectively, in comparison with the uncorrected values of −0.31, 3.59, 2.16, and 0.68 eV. The perturbation of the binding strength hinders the OH removal from the Ir active site, leading to the uphill of the Δ*G*_max_ from 0.22 to 0.30 eV. Despite the activity decrease, the 4*e*^−^ reduction pathway remains, supported by the values of Δ*G*_2a_ with −0.23 eV and Δ*G*_2b_ with 1.26 eV. Herein, the same phenomenon is found for Co_3_(HITP)_2_ and Rh_3_(HITP)_2_ with the Δ*G*_max_ of 0.68 and 0.59 eV, respectively. The corresponding ORR activity with th 4*e*^−^ reaction mechanism follows the order of Ir > Rh ≈ Co. Despite the numerical variation, the trend is roughly consistent with the results without considering vdW interaction.

**Table 1:** The *E*_ads_ and Δ*G* of ORR intermediates at the potential *U* = 0.4V with and without the vdW corrections.

	**Without vdW**			**With vdW**		
	**Co**	**Rh**	**Ir**	**Co**	**Rh**	**Ir**
*E*_ads_ (O_2_)	−0.64	−0.41	−0.31	−0.67	−0.50	−0.39
*E*_ads_ (OOH)	3.42	3.52	3.59	3.20	3.41	3.49
*E*_ads_ (O)	2.53	2.59	2.16	2.54	2.55	2.11
*E*_ads_ (OH)	0.46	0.61	0.68	0.28	0.54	0.61
Δ*G*_1_	−0.08	−0.12	−0.06	−0.31	−0.23	−0.16
Δ*G*_2a_	0.03	0.13	−0.29	0.26	0.20	−0.23
Δ*G*_2b_	1.18	1.21	1.15	1.40	1.32	1.26
Δ*G*_3_	−0.44	−0.53	0.12	−0.64	−0.56	0.09
Δ*G*_4_	0.50	0.52	0.22	0.68	0.59	0.30
Δ*G*_max_	0.50	0.52	0.22	0.68	0.59	0.30

Despite the presence of the Ni_3_(HITP)_2_ experimentally, other 2D catalysts are only theoretical models, which need the confirmation of their synthesis. It should be noted that changing metal atoms would significantly modify the corresponding structures. Thus, by replacing the Ni central atom, the dimensionality of the Cr_3_(HITP)_2_ could be transferred from 2D to 3D due to the energetic favorable insertion of the spacer linker (Foster et al., 2016). Different structures inevitably lead to distinct catalytic performances (Sun et al., 2014). In this regard, although the theoretical candidates have been rationally predicted, the ORR performance of the optimum TM_3_(HITP)_2_ with the Co/Rh/Ir active sites crucially needs further experimentally verification.

## Conclusion

Based on the DFT, the ORR mechanisms on TM_3_(HITP)_2_ monolayer have been studied. It is found that the selection of central metals affects the adsorption behaviors, tuning ORR activity and its selectivity. It is realized that the adsorption abilities are monotonously enhanced as the *d* band upshifts from group 10 to group 8. A classical volcano relationship for the predicted ORR activity as a function of calculated OH adsorption energy is observed. From the calculated Δ*G* data, the ORR activity decreases in the order of Ir > Co ≈ Rh > Ni ≈ Pd > Pt ≈ Fe > Ru > Os. That is, group 9 possesses superior activity compared with other elements. Furthermore, owing to the insufficient O_2_ activation, the 2*e*^−^ mechanism is prevalent in group 10, while the desirable 4*e*^−^ mechanism is dominant for others. These results would throw insights into the nature of the ORR mechanisms of TM_3_(HITP)_2_. The materials of the Co_3_(HITP)_2_, Rh_3_(HITP)_2_, and Ir_3_(HITP)_2_ have been screened out and served as the potential candidates for the ORR electrocatalysis.

## Author contributions

BX carried out the simulation and wrote the paper. QJ revised the paper. HZ, HL, and XJ entered the discussion. All authors commented on the manuscript.

### Conflict of interest statement

The authors declare that the research was conducted in the absence of any commercial or financial relationships that could be construed as a potential conflict of interest.
